# Nitrate from Drinking Water and Diet and Bladder Cancer Among Postmenopausal Women in Iowa

**DOI:** 10.1289/EHP191

**Published:** 2016-06-03

**Authors:** Rena R. Jones, Peter J. Weyer, Curt T. DellaValle, Maki Inoue-Choi, Kristin E. Anderson, Kenneth P. Cantor, Stuart Krasner, Kim Robien, Laura E. Beane Freeman, Debra T. Silverman, Mary H. Ward

**Affiliations:** 1 Occupational and Environmental Epidemiology Branch, Division of Cancer Epidemiology and Genetics, National Cancer Institute, National Institutes of Health (NIH), Department of Health and Human Services (DHHS), Bethesda, Maryland, USA; 2 Center for Health Effects of Environmental Contamination, University of Iowa, Iowa City, Iowa, USA; 3 National Institute on Minority Health and Health Disparities, NIH, DHHS, Bethesda, Maryland, USA; 4 Division of Epidemiology and Community Health, University of Minnesota, Minneapolis, Minnesota, USA; 5 Prevention and Etiology Research Program, Masonic Cancer Center, University of Minnesota, Minneapolis, Minnesota, USA; 6 Metropolitan Water District of Southern California, La Verne, California, USA; 7 Department of Exercise and Nutrition Sciences, Milken Institute School of Public Health, George Washington University, Washington, DC, USA

## Abstract

**Background:**

Nitrate is a drinking water contaminant arising from agricultural sources, and it is a precursor in the endogenous formation of N-nitroso compounds (NOC), which are possible bladder carcinogens.

**Objectives:**

We investigated the ingestion of nitrate and nitrite from drinking water and diet and bladder cancer risk in women.

**Methods:**

We identified incident bladder cancers among a cohort of 34,708 postmenopausal women in Iowa (1986–2010). Dietary nitrate and nitrite intakes were estimated from a baseline food frequency questionnaire. Drinking water source and duration were assessed in a 1989 follow-up. For women using public water supplies (PWS) > 10 years (n = 15,577), we estimated average nitrate (NO3-N) and total trihalomethane (TTHM) levels and the number of years exceeding one-half the maximum contaminant level (NO3-N: 5 mg/L, TTHM: 40 μg/mL) from historical monitoring data. We computed hazard ratios (HRs) and 95% confidence intervals (CIs), and assessed nitrate interactions with TTHM and with modifiers of NOC formation (smoking, vitamin C).

**Results:**

We identified 258 bladder cancer cases, including 130 among women > 10 years at their PWS. In multivariable-adjusted models, we observed nonsignificant associations among women in the highest versus lowest quartile of average drinking water nitrate concentration (HR = 1.48; 95% CI: 0.92, 2.40; ptrend = 0.11), and we found significant associations among those exposed ≥ 4 years to drinking water with > 5 mg/L NO3-N (HR = 1.62; 95% CI: 1.06, 2.47; ptrend = 0.03) compared with women having 0 years of comparable exposure. TTHM adjustment had little influence on associations, and we observed no modification by vitamin C intake. Relative to a common reference group of never smokers with the lowest nitrate exposures, associations were strongest for current smokers with the highest nitrate exposures (HR = 3.67; 95% CI: 1.43, 9.38 for average water NO3-N and HR = 3.48; 95% CI: 1.20, 10.06 and ≥ 4 years > 5 mg/L, respectively). Dietary nitrate and nitrite intakes were not associated with bladder cancer.

**Conclusions:**

Long-term ingestion of elevated nitrate in drinking water was associated with an increased risk of bladder cancer among postmenopausal women.

**Citation:**

Jones RR, Weyer PJ, DellaValle CT, Inoue-Choi M, Anderson KE, Cantor KP, Krasner S, Robien K, Beane Freeman LE, Silverman DT, Ward MH. 2016. Nitrate from drinking water and diet and bladder cancer among postmenopausal women in Iowa. Environ Health Perspect 124:1751–1758; http://dx.doi.org/10.1289/EHP191

## Introduction

Urinary bladder cancer is the sixth most common malignancy in the United States, with an incidence among men that is 4 times greater than that in women [National Cancer Institute Surveillance, Epidemiology, and End Results Program (SEER) 2015]. Established risk factors include cigarette smoking (Silverman et al. 2006), certain occupational exposures (Silverman et al. 2006), and ingestion of high levels of arsenic in drinking water [International Agency for Research on Cancer (IARC) 2012; Saint-Jacques et al. 2014]. Increased exposure to disinfection by-products (DBP) in public drinking water supplies has also been associated with increased bladder cancer risk in studies in North America and Europe (Cantor 1997; Cantor et al. 2006; Costet et al. 2011). The relationship between bladder cancer risk and other drinking water contaminants has not been as well studied.

Nitrate is a common drinking water contaminant arising primarily from agricultural sources, such as nitrogen fertilizers and manure and human waste. In the United States, agricultural areas often have elevated levels of nitrate in ground water and in surface waters (Nolan and Stoner 2000; Ward 2009). Nitrate is also found at high levels in certain vegetables. Nitrite sources in the U.S. diet include processed meats, breads, and cereals (IARC 2010). Ingestion of nitrate and nitrite can lead to endogenous formation of *N*-nitroso compounds (NOC) in the presence of nitrosatable precursors such as amines and amides from meat and fish. NOC also have exogenous sources, including cigarette smoke and meats preserved with nitrite and nitrate salts (IARC 2010).

Ingestion of nitrate and nitrite under conditions that result in the endogenous formation of NOC is classified as a probable human carcinogen by the International Agency for Research on Cancer (IARC 2010) based on animal and mechanistic studies and on limited epidemiologic evidence for stomach and esophagus cancers. Animal data support a role for NOC in causing tumors in numerous organ sites, including the bladder (IARC 2010). In human biomonitoring studies, simultaneous ingestion of nitrate in drinking water at the World Health Organization (WHO) acceptable daily dietary intake level (3.7 mg/kg; WHO 2011) and an amine source resulted in excretion of NOC in the urine (IARC 2010; Vermeer et al. 1998). Intragastric nitrosation is inhibited by antioxidants, including vitamins C and E (IARC 2010; Mirvish 1986), but it is unclear to what extent modification of endogenous NOC formation occurs outside the gastrointestinal tract.

Despite the carcinogenic potential of NOC, nitrate in drinking water has not been extensively evaluated in relation to bladder cancer risk. Case–control studies in Iowa (Ward et al. 2003) and Spain (Espejo-Herrera et al. 2015) found no association between long-term average nitrate levels in public water supplies (PWS) and bladder cancer. Findings from two prospective studies include a significant positive association in the Iowa Women’s Health Study (IWHS) (Weyer et al. 2001) and no association in a Dutch cohort (Zeegers et al. 2006). The Spanish study accounted for concomitant exposure to DBP, which are suspected bladder carcinogens, by adjusting for estimated total trihalomethanes (TTHM) in PWS (Espejo-Herrera et al. 2015). To our knowledge, no U.S. study of drinking water nitrate and bladder cancer has accounted for potential confounding by DBP.

In contrast to the inconclusive findings for drinking water nitrate, positive associations between bladder cancer risk and dietary sources of nitrate and nitrite have been identified in three prospective cohorts (Ferrucci et al. 2010; Michaud et al. 2006) and in two case–control studies (Catsburg et al. 2014; Wu et al. 2012). These studies have implicated intake of processed meats or their nitrate/nitrite preservatives as risk factors, but only the studies by Catsburg et al. (2014) and Ferrucci et al. (2010) explored potential interactions with dietary antioxidants.

In the present study, we expanded a prior analysis of drinking water nitrate concentrations in PWS and bladder cancer risk in the IWHS cohort that included 47 cases (Weyer et al. 2001). With an additional 12 years of follow-up, 130 cases with > 10 years at their public water source, and an improved drinking water exposure assessment, we estimated the bladder cancer risk associated with drinking water nitrate, adjusting for TTHM levels. We also evaluated dietary sources of nitrate and nitrite and assessed whether associations were modified by factors that may inhibit or promote endogenous nitrosation.

## Methods

### Study Population and Case Ascertainment

Details about the IWHS are available elsewhere (Folsom et al. 1989). Briefly, in 1986, a questionnaire was mailed to 98,030 women 55–69 years old who were randomly selected from Iowa driver’s license records. The baseline survey included questions on demographics, anthropometry, reproductive and medical history, tobacco and alcohol consumption, diet, physical activity, and family history of cancer. Informed consent was implied by participants returning the enrollment survey; a total of 41,836 (42%) women responded. The original study and the present analyses were approved by the Institutional Review Boards (IRBs) of the University of Minnesota and the University of Iowa. The present analyses were also approved by the Office of Human Subjects Research at the National Cancer Institute and were considered exempt from IRB review.

A 1989 follow-up questionnaire was completed by 36,127 (87.2%) participants, who indicated the primary source of drinking water at their current residence (municipal water system, rural water system, private well, bottled water, other, don’t know) and the length of time they drank from this source (< 1 year, 1–5 years, 6–10 years, 11–20 years, > 20 years, don’t know). A majority of women (76.7%) reported a public (municipal or rural) source, 18.5% used a private well, and less than 5% reported using bottled water or other sources. Ninety percent reported using their water source for > 10 years; we limited our evaluation to these women.

We identified incident urinary bladder cancers diagnosed between 1 January 1986 and 31 December 2010 by linkage with the State Health Registry (SHR) of Iowa (http://www.public-health.uiowa.edu/shri/) and assessed diagnosis date, topography, morphology, and stage. Participant vital status for censoring purposes was determined annually through linkages with the National Death Index [National Center for Health Statistics (NCHS) 2015] and the SHR. Person-years of follow-up were calculated from the date of enrollment until the earliest of the following dates: incident bladder cancer diagnosis, death, emigration from Iowa (< 0.5% annually), the midpoint between the date of last contact and the date the subject was located outside of Iowa, or 31 December 2010. For deaths outside of Iowa, we censored participants at the midpoint between the date of last contact and death.

### Exposure Assessment

***Drinking water***. The exposure assessment for nitrate in drinking water has been described (Inoue-Choi et al. 2015; Weyer et al. 2001). We used historical monitoring data (1955–1988) for finished water samples from Iowa public water utilities to compute annual average levels of nitrate-nitrogen [milligrams/liter NO_3_-N; Center for Health Effects of Environmental Contamination (CHEEC) 2013; data available upon request]. Direct measurements of TTHM were sparse before implementation of their regulation; therefore, most concentrations before 1980 were estimates based on an expert assessment using measurements and information on water source, treatment practices, and other factors (Amy et al. 2005). We obtained estimates of historical levels of two specific THM (chloroform, bromodichloromethane), TTHM (the sum of chloroform, bromoform, bromodichloromethane, and dibromochloromethane), three specific haloacetic acids (HAA; trichloroacetic, dichloroacetic, and bromochloroacetic acid), HAA5 (the sum of trichloroacetic, dichloroacetic, and monochloroacetic acids), and HAA6 (the sum of HAA5 and bromochloracetic acid); there were few to no occurrence data for other DBP in Iowa. Average levels of chloroform and bromodichloromethane were highly correlated with each other and with TTHM concentration (ρ = 0.95–0.98; see Table S1), and TTHM were correlated with the individual and summed HAA (ρ = 0.79–0.92); we therefore used TTHM levels to represent DBP exposure. TTHM were modestly correlated with annual average nitrate concentrations (ρ = 0.24). Because we had only a categorical duration at the water source as reported on the questionnaire, we estimated the medians within duration categories of 11–20 and > 20 years as 16 and 40 years, respectively. These values were computed from complete water source histories of female controls of comparable age in a population-based case–control study in Iowa conducted during the same time period (Cantor et al. 1998). For each PWS for which both NO_3_ levels and DBP estimates were available, we computed 16- and 40-year averages from the annual averages of NO_3_-N and TTHM levels, as well as the number of years within these periods when the annual average exceeded one-half the maximum contaminant level (½-MCL; NO_3_-N, 5 mg/L and TTHM, 40 μg/mL). We also examined two indicators of DBP presence, including whether a PWS was treated by chloramination (yes/no), a disinfection process that may result in *N*-nitrosodimethylamine (NDMA) formation (Krasner et al. 2013; Mitch et al. 2003), and if it was sourced from surface or ground water. We linked the PWS-level drinking water metrics to participants by the 477 Iowa cities where women reported using PWS.

***Diet.*** Dietary intakes were assessed at baseline using an adaptation of the Harvard semiquantitative food frequency questionnaire (FFQ; Willett et al. 1988), which queried participants regarding their usual intake of 127 food items and dietary supplements over the prior 12 months. A readministration of the FFQ 2 years after enrollment in a small sample of the cohort indicated good reproducibility in measuring key macro- and micronutrients, including total energy intake (*r* = 0.51–0.67) and vitamin C (*r* = 0.81–0.84) (Munger et al. 1992). Total vitamin C intake was calculated by multiplying the frequency of consumption of foods and dietary supplements by their vitamin C content and summing across all foods and supplements.

As described in detail previously (Ward et al. 2003, 2006), we reviewed published data for U.S. and Canadian populations to identify the nitrate and nitrite contents of foods and estimated food-specific mean nitrate and nitrite values, accounting for the preparation method (e.g., raw, cooked, canned) and weighting by the number of samples analyzed. To assign the food-specific nitrate and nitrite levels to the IWHS FFQ line items (e.g., raw carrots), we weighted the contributing food-specific values (e.g., raw carrots, cooked carrots) by female-specific intakes of each food from the 1994–1996 Continuing Survey of Food Intake by Individuals (Subar et al. 2000). For each participant, we estimated overall dietary intakes of nitrate and nitrite and source-specific intakes from plant, animal, and processed meat (e.g., sausage, salami, bologna, bacon, hot dogs) sources.

### Statistical Analysis

Of the 41,836 women enrolled in the IWHS, we excluded those who reported a prior cancer (other than nonmelanoma skin cancer) or previous cancer chemotherapy (*n* = 3,830), had implausible dietary intakes (< 600 or > 5,000 kcal/day) or were missing responses to ≥ 30 dietary questions (*n* = 2,751), or were still menstruating at enrollment (*n* = 547), to be consistent with the cohort’s target composition of postmenopausal women. After these exclusions, made to preserve consistency with the previous study by Weyer et al. (2001), data for 34,708 women were available for dietary analyses.

For drinking water analyses, we further excluded women who reported using their water source for ≤ 10 years in 1989 (*n* = 5,168 on PWS, *n* = 624 on private wells) and those not reporting a duration (*n* = 4,705 on PWS, *n* = 13 on private wells). We also excluded women on PWS with no nitrate or TTHM measurements during the time period they were drinking from the PWS (*n* = 1,638). To reduce variability in contaminant levels within a PWS because of changes in its sources over time, we excluded women on supplies that either lacked detail on sources or that had a sole surface water source or aquifer for < 75% of the study period (< 30 years; *n* = 1,615). These exclusions left 15,910 women on PWS and 5,035 women on private wells for analysis.

We used Cox regression to estimate hazard ratios (HRs) and 95% confidence intervals (95% CIs). In drinking water analyses, we compared average nitrate exposure quartiles with the lowest exposure category (Q1). We divided years > ½-MCL at the median (4 years) and compared with those having no years of exposure at this level. We evaluated the linearity of these relationships by modeling the exposures as continuous variables and by including cubic splines and quadratic exposure terms. The nonparametric analyses showed no statistically significant evidence of nonlinear relationships (data not shown), so we present only natural log–transformed (ln-transformed) results for continuous models in addition to those from categorical analyses. Because no measurement data were available for private wells, we compared women on private well water with women on PWS in Q1 of average nitrate exposure. In dietary analyses, we estimated relative risks for quartiles of dietary nitrate and nitrite overall and by nitrite intakes from plant, animal, and processed meat sources. We evaluated a number of potential confounders ascertained from the baseline questionnaire in models of both diet and water exposures. Covariates were selected for inclusion in a stepwise fashion and were retained based on a ≥ 10% change in the effect estimate and included sociodemographic and lifestyle characteristics and history of various site-specific cancers among first-degree female relatives. Smoking status (never, former, current smoker) was assessed at baseline and in the 1992, 1997, and 2004 follow-up surveys, with participation rates of 82.6%, 79.1%, and 68.3%, respectively. We modeled the most recent smoking status available and pack-years of smoking (0, ≤ 1 to 19, 20 to 39, ≥ 40) reported at baseline, the only survey in which both smoking intensity and duration were assessed. We evaluated separate adjustments for smoking intensity and duration in all models. We computed age-adjusted associations (Model 1), ran multivariable models further adjusted for smoking status and baseline pack-years (Model 2), and then additionally adjusted these models for ln-transformed TTHM concentration (Model 3). Dietary models were adjusted for smoking status, age, and total calorie intake, and mutually adjusted for ln-transformed continuous dietary nitrate or nitrite intake.

We tested for linear trends by modeling continuous variables derived from the median value within each exposure category. We assessed effect modification by TTHM (< median or ≥ median) and smoking status (never, former, or current) by estimating HRs relative to common jointly low-exposed reference groups of those with the lowest levels of average nitrate or 0 years > 5 mg/L and the lowest level of the modifier (< median TTHM and nonsmokers, respectively). The common reference group for vitamin C analyses was low nitrate and ≥ median vitamin C, the group of women with the *a priori*–assumed lowest risk, given that vitamin C is known to inhibit intragastric nitrosation under certain conditions. We used likelihood ratio tests comparing the fit of models with and without product interaction terms to derive an overall interaction *p*-value for each potential effect modifier.

We used several approaches to assess consistency with prior investigations and sources of bias. The initial analysis by Weyer et al. (2001) was restricted to utilities where ≥ 90% of the water in the supply came from a single source. Because we relaxed this criterion to ≥ 75%, we compared our results with risks estimated using the original criterion. The U.S. Environmental Protection Agency’s (EPA’s) 1992 Phase II rule minimally requires annual nitrate monitoring under the Safe Drinking Water Act (U.S. EPA 1991). However, monitoring frequency prior to this rule depended on the size of the population served by the PWS, and additional testing may have been implemented in areas where previous measurements were elevated (U.S. EPA 2012); therefore, our nitrate exposure metrics were derived from a variable number of measurements per PWS. We computed the coefficient of variation (CV) between years for annual mean nitrate levels and repeated the analyses after excluding women in the top 10% of between-year exposure variation. To further evaluate the influence of data availability on our results, we also repeated the analyses after restricting to women with long duration at their water source (> 20 years) whose average nitrate level was based on at least the median number of years of data available (≥ 8 years). We conducted all analyses in SAS v.9.3 (SAS Institute Inc.) with *p* ≤ 0.05 as the criterion for statistical significance.

## Results

We observed 263 urinary bladder cancers among 34,708 postmenopausal women over an average of 21 years (median = 25 years) of follow-up, including 170 cases among the women who used a private well (*n* = 5,035) or PWS (*n* = 15,910) as their primary drinking water source > 10 years. Most women with private wells lived on a farm or in nonfarm rural areas, whereas most PWS users lived in towns (Table 1). The fraction of women on PWS sourced from surface water varied by nitrate quartile, with 46% in the highest category. Conversely, women with the highest average PWS nitrate levels had the lowest proportion of PWS disinfected by chloramination. Other factors did not differ appreciably by nitrate level. Compared with private well users, current smoking was more prevalent among PWS users, who also tended to have lower BMI, consume fewer calories, and were more highly educated. Average nitrate and TTHM levels varied across the 10 most populated cities and towns in our analysis (37% of the cohort using PWS) with no pattern, and the most populated city, Des Moines, accounted for only 8% of the women in our analysis (see Table S2).

**Table 1:** Characteristics of Iowa Women’s Health Study participants with > 10 years at their drinking water source, by private well use and nitrate-nitrogen (NO_3_-N) levels in public water.

Characteristic	Private well (*n *= 5,035)	Mean^*a*^ NO_3_-N (mg/L) levels in public water (*n *= 15,910)			
		< 0.47	0.47–1.07	1.08–2.97	> 2.97
Length of follow-up, years (mean ± SD)	21.3 ± 5.5	20.7 ± 5.9	20.5 ± 5.9	20.6 ± 5.9	20.6 ± 6.0
Age at baseline, years (mean ± SD)	61.2 ± 4.1	61.7 ± 4.2	61.7 ± 4.2	61.6 ± 4.2	61.6 ± 4.2
White race [*n* (%)]	4,971 (99.6)	3,999 (99.5)	3,849 (99.2)	4,146 (99.2)	3,645 (98.7)
Missing (*n*)	46	46	34	31	29
Surface water as source for PWS [*n* (%)]	—	213 (5.2)	1,101 (28.4)	668 (15.9)	1,716 (46.1)
PWS chloraminated [*n* (%)]	—	721 (17.7)	1,483 (37.9)	1,614 (38.3)	273 (7.3)
Nitrate in diet, mg/day (median)^*b*^	59.1	61.2	61.3	61.8	62.0
Nitrite in diet, mg/day (median)^*b*^	0.66	0.64	0.64	0.65	0.65
Vitamin C in diet, mg/day (median)^*b*^	99.9	108.0	111.8	108.7	112.9
Total caloric intake, kcal/day (median)	1,829	1,696	1,683	1,693	1,682
Smoking status^*c*^ [*n* (%)]
Never	3,882 (78.3)	2,499 (62.4)	2,393 (61.7)	2,487 (59.7)	2,275 (62.1)
Former	850 (17.2)	1,174 (29.3)	1,157 (29.7)	1,329 (31.9)	1,079 (29.4)
Current	223 (4.5)	334 (8.3)	325 (8.4)	347 (8.3)	311 (8.4)
Missing (*n*)	80	57	38	48	57
Pack-years of smoking^*d*^ [*n* (%)]
1–19	468 (45.0)	594 (40.6)	604 (41.5)	624 (38.2)	526 (39.1)
20–39	358 (34.4)	488 (33.3)	489 (33.6)	576 (35.2)	461 (34.3)
≥ 40	214 (20.6)	383 (26.1)	363 (24.9)	435 (26.6)	358 (26.6)
Missing (*n*)	25	34	22	33	44
Occupation [*n* (%)]
Homemaker	2,643 (52.5)	1,322 (32.5)	1,309 (33.5)	1,366 (32.4)	1,221 (32.8)
Professional	604 (12.0)	670 (16.5)	657 (16.8)	636 (15.1)	618 (16.6)
Clerical/craft	1,293 (25.7)	1,995 (49.1)	1,883 (48.1)	2,139 (50.8)	1,834 (49.3)
Farmer	473 (9.4)	60 (1.5)	46 (1.2)	41 (1.0)	34 (0.9)
Other	22 (0.4)	17 (0.4)	18 (0.5)	41 (0.7)	34 (0.4)
Residence [*n *(%)]
Farm	3,592 (71.5)	129 (3.2)	129 (3.3)	90 (2.2)	98 (2.7)
Rural area (nonfarm)	971 (19.3)	73 (1.8)	86 (2.2)	61 (1.5)	106 (2.9)
Towns ≥ 1,000 residents	460 (9.2)	3,844 (95.0)	3,660 (94.5)	4,035 (96.4)	3,494 (94.5)
Missing (*n*)	12	18	38	25	24
BMI (kg/m^2^) [*n* (%)]
< 25	1,797 (35.7)	1,658 (40.8)	1,661 (42.5)	1,801 (42.8)	1,551 (41.7)
25–29.9	1,914 (38.0)	1,514 (37.3)	1,418 (36.2)	1,511 (35.9)	1,364 (36.7)
≥ 30	1,324 (26.3)	892 (22.0)	834 (21.3)	899 (21.4)	807 (21.7)
Education [*n* (%)]
Less than high school	505 (10.1)	275 (6.8)	237 (6.1)	276 (6.6)	202 (5.4)
High school	2,621 (52.2)	2,163 (53.3)	2,030 (52.0)	2,268 (54.0)	1,828 (49.1)
More than high school	1,900 (37.8)	1,619 (39.9)	1,640 (42.0)	1,658 (39.5)	1,690 (45.4)
Missing (*n*)	9	7	6	9	2
Abbreviations: BMI, body mass index; PWS, public water supplies; SD, standard deviation. ^***a***^Exposure assigned to individuals based on their reported duration at their drinking water source. ^***b***^Adjusted for 1,000 kcal per day of total energy intake. ^***c***^Determined based on most recent follow-up participation; otherwise from baseline report. ^***d***^Among current or former smokers at baseline.					

In multivariable-adjusted models, women in the highest quartile of mean drinking water nitrate exposure had a nonsignificantly higher relative risk of bladder cancer (Model 2 HR_Q4 vs. Q1_ = 1.48, 95% CI: 0.92, 2.40; *p*_trend_ = 0.11); the association was very similar following additional adjustment for TTHM (Model 3; Table 2) and for chloramination status (HR_Q4 vs. Q1_ = 1.43, 95% CI: 0.87, 2.33). The risk among women exposed to ≥ 4 years above the ½-MCL level was significantly greater than for women with no years of exposure to levels above this threshold (Model 2 HR = 1.62, 95% CI: 1.06, 2.47; *p*_trend_ = 0.03), and associations were also similar after further adjustment for TTHM (Model 3; Table 2). Positive associations were also present with exposures analyzed as continuous variables, significantly so for the ½-MCL metric (Table 2). We observed no association in multivariable-adjusted models comparing *n* = 4,930 private well users (*n* = 36 cases) with women on PWS in the lowest NO_3_-N quartile (Model 2 HR = 1.16, 95% CI: 0.70, 1.91). We observed no significant associations with TTHM and bladder cancer for either the TTHM long-term average (Model 2 HR_Q4 vs. Q1_ = 0.93, 95% CI: 0.55, 1.58) or the ½-MCL exposure metric (Model 2 HR_≥ 4 years vs. 0_ = 0.80, 95% CI: 0.47, 1.38).

**Table 2:** Association between drinking water nitrate-nitrogen (NO_3_-N) in public water supplies and bladder cancer in the Iowa Women’s Health Study (*n *= 15,577*^a^*).

Drinking water nitrate	Cases	*n*	Model 1^*b*^ HR (95% CI)	Model 2^*c*^ HR (95% CI)	Model 3^*d*^ HR (95% CI)
Average NO_3_-N (mg/L)
< 0.47	29	3,973	1.00 (Reference)	1.00 (Reference)	1.00 (Reference)
0.47–1.07	32	3,853	1.16 (0.70, 1.91)	1.16 (0.70, 1.92)	1.14 (0.68, 1.90)
1.08–2.97	30	4,130	1.00 (0.60, 1.67)	0.98 (0.59, 1.64)	0.97 (0.58, 1.62)
> 2.97	39	3,621	1.49 (0.92, 2.41)	1.48 (0.92, 2.40)	1.47 (0.91, 2.38)
*p*_trend_^*e*^			0.10	0.11	0.11
Continuous^*f*^	130	15,577	1.13 (0.96, 1.32)	1.12 (0.95, 1.32)	1.12 (0.95, 1.32)
Years ½-MCL (> 5 mg/L NO_3_-N)
0	83	10,947	1.00 (Reference)	1.00 (Reference)	1.00 (Reference)
< 4	18	2,295	1.04 (0.62, 1.73)	1.05 (0.63, 1.75)	1.06 (0.63, 1.76)
≥ 4	29	2,335	1.66 (1.09, 2.53)	1.62 (1.06, 2.47)	1.61 (1.05, 2.47)
*p*_trend_^*e*^			0.02	0.03	0.03
Continuous^*g*^	130	15,577	1.07 (1.01, 1.13)	1.06 (1.00, 1.12)	1.06 (1.00, 1.12)
^***a***^After excluding 333 women with missing covariate data. ^***b***^Adjusted for age. ^***c***^Adjusted for age, smoking status, and pack-years of smoking. ^***d***^Adjusted for age, smoking status, pack-years of smoking, and ln-transformed total trihalomethane (TTHM) level. ^***e***^Estimated by modeling a continuous variable derived from the median value within each exposure category. ^***f***^Hazard ratio (HR) per 1 natural log increase in concentration (milligrams/liter). ^***g***^HR per 1 year increase in number of years > ½-MCL. Abbreviations: ½-MCL, one-half the maximum contaminant level; CI, confidence interval.					

Our sensitivity analyses, which applied a more conservative source criterion (≥ 90% of the PWS served from the same source, *n* = 14,775, 121 cases) or excluded women whose nitrate exposures represented the top 10% of the variance across estimates (*n* = 14,029, 110 cases), did not materially change these associations (data not shown). We observed somewhat stronger associations among the subset of women with > 20 years of use of their PWS whose average nitrate exposure was estimated from 8 or more years of measurements (*n* = 8,032; Model 2 HR_Q4 vs. Q1_ = 2.38, 95% CI: 1.08, 5.22; *p*_trend_ = 0.02; see Table S3); the number of years of available data was weakly correlated with nitrate levels (ρ = 0.11).

Nitrate models stratified by smoking status suggested a multiplicative interaction (global *p*-value for interaction = 0.03) on bladder cancer risk (Table 3), with the strongest association among current smokers consuming the highest average nitrate level compared with never smokers having low average nitrate levels (HR = 3.67, 95% CI: 1.43, 9.38; *p*_interaction_ = 0.03). We observed a somewhat similar pattern for the ½-MCL exposure metric (*p*_interaction_ = 0.01), with the strongest association among current smokers with ≥ 4 years drinking water at > ½-MCL, as well as elevated relative risks among former smokers, compared with never smokers with no years > ½-MCL.

**Table 3:** Association between drinking water nitrate-nitrogen (NO_3_-N) in public water supplies and bladder cancer in the Iowa Women’s Health Study, stratified by smoking status (*n *= 15,577).

Drinking water nitrate	Never smokers			Former smokers			Current smokers			*p*_interaction_^*b*^
	Cases	*n*	HR^*a*^ (95% CI)	Cases	*n*	HR^*a*^ (95% CI)	Cases	*n*	HR^*a*^ (95% CI)	
Average NO_3_-N (mg/L)
< 0.47	17	2,499	1.00 (Reference)	8	1,149	0.70 (0.28, 1.76)	4	325	1.24 (0.37, 4.13)
0.47–1.07	13	2,393	0.81 (0.39, 1.67)	16	1,142	1.43 (0.66, 3.14)	3	318	1.00 (0.26, 3.78)
1.08–2.97	14	2,487	0.83 (0.41, 1.68)	11	1,305	0.83 (0.35, 1.95)	5	338	1.57 (0.51, 4.82)
> 2.97	15	2,275	0.97 (0.49, 1.95)	14	1,048	1.30 (0.58, 2.94)	10	298	3.67 (1.43, 9.38)	0.03
Total	59	9,654		49	4,644		22	1,279
Years > ½–MCL (> 5 mg/L NO_3_-N)
0	39	6,784	1.00 (Reference)	34	3,268	1.25 (0.68, 2.27)	10	895	1.39 (0.59, 3.30)
< 4	9	1,475	1.06 (0.51, 2.19)	2	631	0.38 (0.09, 1.67)	7	189	4.69 (1.79, 12.27)
≥ 4	11	1,395	1.39 (0.71, 2.71)	13	745	1.99 (0.94, 4.22)	5	195	3.48 (1.20, 10.06)	0.01
Total	59	9,654		49	4,644		22	1,279
Abbreviations: ½-MCL, one-half the maximum contaminant level; CI, confidence interval; HR, hazard ratio. ^***a***^Adjusted for age and pack-years of smoking. ^***b***^Derived from a likelihood ratio test comparing fit of models with and without a cross-product term for smoking status and nitrate exposure.										

In models stratified by TTHM level, we observed a nonsignificant positive association among women drinking from PWS with high average nitrate (> 2.97 mg/L) and ≥ median TTHM levels (HR_Q4 vs. Q1_ = 1.64, 95% CI: 0.94, 2.86) compared with women < median TTHM and low NO_3_-N, but not among women with < median TTHM and high NO_3_-N (HR_Q4 vs. Q1_ = 1.12, 95% CI: 0.53, 2.37; *p*_interaction_ = 0.07; see Table S4). We found little evidence for interaction between either PWS nitrate exposure metric and vitamin C intake (see Table S5). Compared with those having the lowest average intake of NO_3_-N and ≥ median vitamin C, we observed associations among women with high NO_3_-N and < median vitamin C (HR = 3.05, 95% CI: 1.37, 6.79) as well as elevated risk among women with high NO_3_-N and > median vitamin C (*p*_interaction_ = 0.27; see Table S5). Similar patterns were observed with the ½-MCL metric. However, we observed a significant positive association with private well use and bladder cancer among women with ≥ median vitamin C intake compared with women on PWS with low average nitrate levels (HR = 2.38, 95% CI: 1.03, 5.51). In contrast, there was no association with private well use among women with < median vitamin C intake (HR = 0.69, 95% CI: 0.36, 1.35; *p*_interaction_ = 0.02).

Dietary nitrate intake came almost exclusively from plant sources (median proportion = 97.0%); thus, only total nitrate is presented, whereas nitrite came from both plant (62.3%) and animal (37.7%) sources, including 4.4% from processed meats. Nitrate and nitrite intakes overall were moderately correlated (ρ = 0.49) and were strongly correlated for processed meats (ρ = 0.99). We observed no overall association between dietary nitrate intake and bladder cancer risk, and no association for dietary nitrite overall or from separate sources (Table 4). There was no pattern of different risk estimates by vitamin C intake group when comparing with common reference groups of ≥ median vitamin C and either low nitrate or nitrite, and global tests for interaction were not statistically significant (see Table S6). Compared with a common reference group of never smokers in Q1 for dietary nitrite intake, the risk of bladder cancer among current smokers with the highest nitrite intake was significantly increased (HR = 2.66, 95% CI: 1.05, 6.75), whereas there was a nonsignificant positive association among former smokers (HR = 1.49, 95% CI: 0.70, 3.21) and no association with high nitrite intake among never smokers (HR = 0.75, 95% CI: 0.38, 1.49; *p*_interaction_ = 0.06; see Table S7). Nonsignificant associations with dietary nitrate intake were apparent across intake quartiles among current smokers only (*p*_interaction_ = 0.10; see Table S7).

**Table 4:** Association between dietary nitrate and nitrite and bladder cancer in the Iowa Women’s Health Study (*N *= 33,964*^a^*).

Nitrate/nitrite sources	Cases	*n*	Age-adjusted HR (95% CI)	Fully adjusted^*b*^ HR (95% CI)
Dietary nitrate (mg NO_3_-N/day^*c*^)
All sources
< 16.2	67	8,467	1.00 (Reference)	1.00 (Reference)
16.2–23.9	68	8,489	1.00 (0.72, 1.41)	1.01 (0.72, 1.43)
24.0–34.2	64	8,506	0.92 (0.66, 1.30)	0.94 (0.66, 1.35)
> 34.2	59	8,502	0.86 (0.60, 1.22)	0.85 (0.58, 1.26)
*p*_trend_^*d*^			0.32	0.34
Continuous^*e*^	258	33,964	0.85 (0.69, 1.04)	0.83 (0.65, 1.05)
Dietary nitrite (mg/day)
All sources
< 0.86	63	8,450	1.00 (Reference)	1.00 (Reference)
0.86–1.12	66	8,514	1.01 (0.71, 1.42)	1.15 (0.78, 1.70)
1.13–1.43	73	8,487	1.12 (0.80, 1.56)	1.38 (0.89, 2.16)
> 1.43	56	8,513	0.85 (0.60, 1.22)	1.15 (0.65, 2.03)
*p*_trend_			0.44	0.69
Continuous	258	33,964	0.95 (0.70, 1.29)	1.59 (0.89, 2.84)
Plant sources
< 0.51	62	8,461	1.00 (Reference)	1.00 (Reference)
0.51–0.67	75	8,507	1.17 (0.84, 1.64)	1.33 (0.92, 1.91)
0.68–0.90	69	8,490	1.08 (0.76, 1.52)	1.32 (0.88, 1.99)
> 0.90	52	8,506	0.81 (0.56, 1.17)	1.05 (0.64, 1.72)
*p*_trend_			0.15	0.82
Continuous	258	33,964	0.86 (0.66, 1.12)	1.10 (0.74, 1.63)
Animal sources
< 0.29	67	8,474	1.00 (Reference)	1.00 (Reference)
0.29–0.40	54	8,443	0.79 (0.55, 1.14)	0.86 (0.59, 1.25)
0.41–0.56	64	8,533	0.92 (0.66, 1.30)	1.03 (0.70, 1.53)
> 0.56	73	8,514	1.06 (0.76, 1.47)	1.24 (0.79, 1.95)
*p*_trend_			0.53	0.22
Continuous	258	33,964	1.10 (0.86, 1.39)	1.31 (0.93, 1.86)
Processed meats
< 0.01	69	8,718	1.00 (Reference)	1.00 (Reference)
0.01–0.03	75	8,707	1.00 (0.72, 1.40)	0.95 (0.68, 1.33)
0.04–0.06	54	8,107	0.89 (0.63, 1.26)	0.86 (0.60, 1.22)
> 0.06	60	8,432	0.95 (0.67, 1.33)	0.88 (0.61, 1.26)
*p*_trend_			0.68	0.46
Continuous	258	33,964	0.99 (0.95, 1.02)	0.98 (0.94, 1.02)
Abbreviations: CI, confidence interval; HR, hazard ratio. ^***a***^After excluding 744 women with missing covariate data. ^***b***^Adjusted for age, smoking status, pack-years of smoking, and ln-transformed total energy intake. Nitrate models were also adjusted for total ln-transformed dietary nitrite from all sources, and nitrite models were adjusted for total ln-transformed dietary nitrate from all sources. ^***c***^NO_3_^–^ converted to NO_3_-N. Nitrate intakes came almost exclusively from plant sources. ^***d***^Estimated by modeling a continuous variable derived from the median value within each exposure category. ^***e***^HR per 1 natural log increase in intake (milligrams/day).				

## Discussion

Consistent with the findings of an earlier report on this cohort (Weyer et al. 2001), we estimated a greater relative risk of bladder cancer among postmenopausal women in Iowa whose drinking water source had higher long-term average nitrate levels, with statistically significant associations for those with 4 or more years at levels > ½-MCL. These associations were generally unchanged by adjustment for TTHM level. Our analyses suggested possible modification of the nitrate-bladder cancer association among women with a history of smoking and among those who were simultaneously exposed to high TTHM levels in their drinking water. Dietary nitrate and nitrite were not associated with bladder cancer. Vitamin C, a known inhibitor of intragastric NOC formation, did not significantly modify the observed associations.

Our finding of a bladder cancer association with long-term average drinking water nitrate exposure is generally consistent with the findings from a previous analysis in the IWHS, which found positive associations among women who had been at their water source for > 10 years (RR_Q4 vs. Q1_ = 2.43, 95% CI: 1.01, 5.88) based on 47 cases (Weyer et al. 2001). However, the prior exposure assessment assigned a single 33-year average PWS nitrate level to all participants. In our analysis of nearly three times as many cases using a duration-based average exposure, we found a similar pattern but an overall weaker association among women with > 10 years at their water source, which strengthened when we restricted analyses to women with an estimated > 20 years of exposure.

Other epidemiologic evidence from case–control or cohort studies to support this association is limited. A large population-based bladder cancer case–control study in Iowa (*n* = 808 cases) with comparable PWS nitrate exposure levels found no relationship with bladder cancer overall, nor among the 186 female cases specifically (Ward et al. 2003). Another population-based case–control analysis of exposure to chlorinated DBP in Colorado (*n* = 327 cases) reported no association or confounding effects of concurrent nitrate levels (McGeehin et al. 1993). In a Spanish case–control study of > 1,400 cases, average nitrate levels in drinking water were not associated with bladder cancer (Espejo-Herrera et al. 2015). A large prospective study in the Netherlands with > 800 cases also reported no overall or sex-specific associations (Zeegers et al. 2006). The average long-term nitrate level in the Dutch cohort (1.68 mg/L) was comparable to ours (median = 1.1 mg/L). However, their exposure metric was based on samples from a single year, which may not reflect usual exposure over time. Inconsistent associations between drinking water nitrate and bladder cancer risk across studies could also be the result of regional variation in the mixtures and in the relative abundance of water contaminants resulting from differences in disinfection practices (Costet et al. 2011), agricultural inputs, and the prevalence of naturally occurring chemicals. The extent of exposure misclassification and subsequent ability to detect associations may also differ between studies.

The MCL for nitrate in drinking water (10 mg/L NO_3_-N) was established based on evidence of health risks from short-term exposure, such as methemoglobinemia (blue baby syndrome) in infants (U.S. EPA 2012). Bladder cancer development associated with chronic intake of levels below the MCL has only been evaluated in a small number of studies (Cantor et al. 2006; Villanueva et al. 2014). To our knowledge, our finding of significantly increased bladder cancer risk associated with 4 or more years above ½-MCL exposure is the first such epidemiologic finding in a prospective cohort with quantitative exposure assessment. The abovementioned Spanish study similarly reported elevated bladder cancer ORs associated with longer duration of exposure (> 20 years) to higher levels (> 9 mg/L NO_3_-, i.e., > 2 mg/L NO_3_-N) of drinking water nitrate (Espejo-Herrera et al. 2015). Although our categorical drinking water exposure metrics were correlated (ρ = 0.74), only 15% of the women were exposed to both high (Q4; > 2.97 mg/L) average levels and to ≥ 4 years > 5 mg/L, indicating that these metrics capture different features of nitrate exposure.

A key finding of the analysis conducted by Weyer et al. (2001) was a stronger water nitrate–bladder cancer association when models were adjusted for whether a PWS used surface versus ground water sources; the former is a crude surrogate for higher DBP levels. Although DBP, often represented as TTHM, have been associated with bladder cancer (Cantor et al. 2006; Costet et al. 2011; Villanueva et al. 2014), our associations with drinking water NO_3_-N were upheld with adjustment for TTHM, which were not independently associated with bladder cancer. The present study was underpowered to further examine the suggestive finding of stronger nitrate associations for women with simultaneously higher TTHM exposures, but the Spanish study identified a similar risk pattern (Espejo-Herrera et al. 2015). Estimated TTHM levels in our study (median = 4.6 μg/L, interquartile range 0.9–14.3) were generally below the MCL and below levels associated with bladder cancer in other studies (> 5 μg/L; Costet et al. 2011). Nonregulated and therefore nonmeasured DBP, such as NDMA, could also have driven our observed nitrate associations (Richardson et al. 2007), although adjustment for PWS chloramination did not produce meaningful changes in our results.

We found no consistent associations between dietary nitrate overall or nitrite intake from processed meat and bladder cancer. A similar lack of relationship was reported for a prospective European study of > 1,000 bladder cancer cases that examined associations with intakes of red or processed meats or of dietary nitrosamines (Jakszyn et al. 2011). In contrast, unlike in the prospective Nurses’ Health Study (NHS) cohort (*n* = 304 incident cases; Michaud et al. 2006) and in population-based case–control studies in California (*n* = 1,660 incident cases; Catsburg et al. 2014) and New England (*n* = 1,068 incident cases; Wu et al. 2012), we did not find associations with nitrate or nitrite intakes from processed meat sources. Processed meat intake in our cohort was considerably lower than the intakes reported in these studies. This finding may in part reflect lower intakes in women than in men; dietary intakes of certain NOC, such as the rodent bladder carcinogen NDMA, have been reported to be as much as 3 times higher among men (Dich et al. 1996; Jakszyn et al. 2006). Our ability to compare findings is limited because associations for women specifically were reported only for the NHS (Michaud et al. 2006). Catsburg et al. (2014) reported no effect modification by sex, and the study in New England did not report a sex-specific evaluation (Wu et al. 2012).

Human feeding studies have demonstrated endogenous formation of NOC after ingestion of nitrate at or above the MCL (IARC 2010; Vermeer et al. 1999; Vermeer and van Maanen 2001). Approximately 5% of ingested nitrate is reduced to nitrite by bacteria in the mouth; this nitrite can further react with secondary and some tertiary amines or amides to form NOC in the stomach (Mirvish and Ramm 1987). NOC, such as dibutylnitrosamine, are metabolized by the liver and ultimately pass through the urinary bladder to be excreted (Mirvish 1995). Little is known about the influence of intrinsic or extrinsic factors on NOC formation within the bladder. We evaluated interactions between nitrate and vitamin C on bladder cancer risk based on the capacity of antioxidants to inhibit intragastric NOC formation (Bartsch et al. 1993), a mechanism thought to explain the elevated nitrate-associated gastrointestinal malignancies observed among individuals with low vitamin C intake (Dellavalle et al. 2014; IARC 2010; Loh et al. 2011). Our results provide no compelling support for an interaction between vitamin C and nitrate on bladder cancer risk, consistent with the findings of previous studies of drinking water nitrate (Ward et al. 2003; Espejo-Herrera et al. 2015) and with those of two recent analyses that reported no modification of dietary nitrate–bladder cancer associations by vitamin C from diet or supplementation (Ferrucci et al. 2010) or from estimated fruit and vegetable intakes (Wu et al. 2012). The interpretation of an interaction between vitamin C intake and private well use is unclear because we had no quantitative exposure estimates for these women and the precision of the associations was limited.

Cigarette smoking was a potential confounder in our analysis because it is a known risk factor for bladder cancer and a source of NOC exposure. Smoking was independently associated with bladder cancer risk in our data, as reported previously for this cohort (Tripathi et al. 2002). Thiocyanate in cigarette smoke is a potential catalyst for intragastric NOC formation (Preston-Martin and Correa 1989), and greater endogenous nitrosation could explain stronger associations with drinking water nitrate among smokers. However, the results of these interaction analyses should be interpreted with caution because of their small numbers. We assessed smoking status at both baseline and a more recent time period for many women, which likely reduced misclassification of this variable in our analyses. Smoking intensity was moderately but not linearly correlated with average drinking water nitrate concentrations (data not shown), and we saw no change in estimated associations from adjustment for smoking, including in our main models with intensity and duration combined as pack-years, or in analyses separating these characteristics (data not shown). Therefore, residual confounding from smoking is also unlikely to explain the observed risks.

Our study remains among the few prospective analyses of drinking water nitrate exposure and bladder cancer risk. To our knowledge, it is also the first to assess this association in a cohort study with simultaneous assessment of TTHM. Our PWS-level exposure estimates reflected duration-specific average levels as well as the duration of exposure to elevated levels. The residential stability of the cohort (inferred from the large proportion of women reporting > 20 years at their drinking water source) likely reduced drinking water exposure misclassification caused by changes in drinking water sources over time. Sensitivity analyses with more stringent exposure assessment criteria indicated that our results may underestimate the true drinking water nitrate-bladder cancer association.

We acknowledge some limitations to this study, including an inability to adjust for high-risk occupation. Iowa is predominantly agricultural, and unmeasured occupational or environmental exposures could have contributed to the observed associations. Our data suggest that most participants were not employed in occupations likely to have these exposures and that occupational categories did not vary with drinking water nitrate levels. Moreover, measured lifestyle and other characteristics were not different among women in the top categories of average nitrate exposure. Importantly, we lacked a measure of total fluid intake, which would influence exposure to nitrate from drinking water (IARC 2010). Private well users are potentially exposed to nitrate, but not to DBP, in their drinking water. A limitation of our study was the lack of nitrate exposure estimates for the women using private wells, nor did we have information on well depth, which is an important predictor of nitrate concentrations. We addressed other limitations of our exposure assessment, confirming the absence of a correlation between sampling frequency and nitrate concentration and identifying stronger associations with exposure metrics derived from more years of measurements. The latency of bladder cancer following nitrate exposure is unknown. However, findings from studies of arsenic, another drinking water contaminant, suggest that the latency of arsenic-related bladder cancer is ≥ 40 years (Baris et al. 2016; Steinmaus et al. 2013). Thus, although we had no water measurements after 1988, a long latency for bladder cancer and our retrospective assessment should reduce the impact of this lack of exposure information on our results. Misclassification of dietary intakes is also possible owing to our use of an FFQ and to our inability to identify some factors contributing to nitrate levels in vegetables and to NOC formation in processed meats, such as regional variations in growing conditions and in cooking methods, respectively. Further, whether these findings are specific to the postmenopausal status or the homogeneous racial/ethnic makeup of our female study population or to drinking water sources in Iowa should be further evaluated.

## Conclusion

Our results indicate that ingested nitrate from drinking water may play a role in bladder cancer etiology. We assessed both nitrate and TTHM exposures and bladder cancer risk and found that the previously observed associations with drinking water nitrate remained after additional follow-up. We observed an interaction between drinking water nitrate and smoking status on bladder cancer risk that requires replication; we also observed a suggested interaction between nitrate and TTHM that deserves evaluation in future studies. These results should be interpreted in the context of very limited epidemiologic data on drinking water nitrate exposures and bladder cancer risk and with regard to our population of predominantly non-Hispanic white, postmenopausal women.

## Supplemental Material

(211 KB) PDF
