# Supplementary material for: Nitrate from Drinking Water and Diet and Bladder Cancer Among Postmenopausal Women in Iowa
Source: Environ Health Perspect. 2016 Jun 3;124(11):1751–8. doi: 10.1289/EHP191 (PMC5089883; doi:10.1289/EHP191)
Supplement: (211 KB) PDF [file EHP191.s001.acco.pdf]

Environ Health Perspect
DOI: 10.1289/EHP191

Note to readers with disabilities: *EHP* strives to ensure that all journal content is accessible to all readers. However, some figures and Supplemental Material published in *EHP* articles may not conform to 508 standards due to the complexity of the information being presented. If you need assistance accessing journal content, please contact ehp508@niehs.nih.gov. Our staff will work with you to assess and meet your accessibility needs within 3 working days.

Supplemental Material

Nitrate from Drinking Water and Diet and Bladder Cancer among Postmenopausal Women in Iowa

Rena R. Jones, Peter J. Weyer, Curt T. Dellavalle, Maki Inoue-Choi, Kristin E. Anderson, Kenneth P. Cantor, Stuart Krasner, Kim Robien, Laura E. Beane Freeman, Debra T. Silverman, and Mary H. Ward

Table of Contents

Table S1. Spearman correlations (ρ) between average nitrate (mg/L) and disinfection by-products (μg/L) levels among Iowa Women's Health Study participants on public water supplies with >10 years at their drinking water source (N=15,910)

Table S2. Number and percent of Iowa Women's Health Study participants in the public water supply analyses for the 10 most populated cities and towns

Table S3. Association between drinking water nitrate-nitrogen (NO_3-N) in public water supplies and bladder cancer risk in the Iowa Women's Health Study among women with >20 years duration at their water source and nitrate exposure based on ≥ 8 years of data (N=8,032)

Table S4. Association between average drinking water nitrate-nitrogen (NO_3-N) and bladder cancer by median total trihalomethanes (TTHM) level in the Iowa Women's Health Study (N=15,577)

Table S5. Association between private well use (N=4,930) and drinking water nitrate–nitrogen (NO_3-N) levels in public water supplies (N=15,577) and bladder cancer in the Iowa Women's

Health Study, stratified by vitamin C intake

Table S6. Association between dietary nitrate and nitrite and bladder cancer in the Iowa Women's Health Study, stratified by vitamin C intake (N=33,964)

Table S7. Association between dietary nitrate and nitrite and bladder cancer in the Iowa Women's Health Study, stratified by smoking status (N=33,964)

Table S1. Spearman correlations (ρ) between average nitrate (mg/L) and disinfection by-products (µg/L) levels among Iowa Women's Health Study participants on public water supplies with >10 years at their drinking water source (N=15,910)

	**TTHM**	**ChCl3**	**BDCM**	**DCAA**	**TCAA**	**HAA5**	**BCAA**	**HAA6**	**NO_3-N**
**TTHM**		0.98	0.97	0.79	0.92	0.90	0.82	0.90	0.24
**ChCl3**			0.95	0.82	0.90	0.92	0.76	0.88	0.20
**BDCM**				0.72	0.93	0.84	0.82	0.87	0.22
**DCAA**					0.71	0.92	0.67	0.89	-0.02
**TCAA**						0.87	0.82	0.90	0.15
**HAA5**							0.75	0.95	0.06
**BCAA**								0.87	0.29
**HAA6**									0.16
**NO_3-N**									

TTHM, total trihalomethanes; $CHCl_3$, chloroform; BDCM, bromodicholoromethane; DCAA, dichloroacetic acid; TCAA, trichloroacetic acid; HAA5 and HAA6, haloacetic acids; BCAA, bromochloroacetic acid. NO_3-N, nitrate-nitrogen;

Table S2. Number and percent of Iowa Women's Health Study participants in the public water supply analyses for the 10 most populated cities and towns

Water supply	N	%[a]	Average NO_3-N[b]	Average TTHMs[b]
Des Moines	1340	8.42	3.92	14.62
Cedar Rapids	931	5.85	1.13	6.67
Davenport	686	4.31	1.12	91.56
Waterloo	676	4.25	3.00	4.52
Sioux City	571	3.59	0.32	4.26
Dubuque	495	3.11	0.27	10.51
Council Bluffs	345	2.17	0.86	47.87
Clinton	310	1.95	0.14	0.48
Mason City	292	1.84	0.32	1.88
Cedar Falls	288	1.81	3.53	0.61

[a] Reflects the percentage of the total population included in the drinking water analyses.
[b] Average of the duration-specific NO_3-N or TTHM means for the women served by each utility.

Table S3. Association between drinking water nitrate-nitrogen (NO_3-N) in public water supplies and bladder cancer risk in the Iowa Women's Health Study among women with >20 years duration at their water source and nitrate exposure based on ≥ 8 years of data (N=8,032)

			**Model 1**[a]	**Model 2**[b]	**Model 3**[c]
**Drinking water nitrate**	**Cases**	**N**	**HR (95% CI)**	**HR (95% CI)**	**HR (95% CI)**
**Average NO_3-N (mg/L)**					
<0.47	8	1,493	1.00 (Ref.)	1.00	1.00 (Ref.)
0.47-1.07	17	2,124	1.54 (0.66,3.57)	1.50 (0.65,3.47)	1.65 (0.70,3.85)
1.08-2.97	16	2,199	1.35 (0.58,3.15)	1.33 (0.57,3.10)	1.39 (0.59,3.25)
>2.97	28	2,216	2.37 (1.08,5.20)	2.38 (1.08,5.22)	2.46 (1.12,5.40)
p_{trend}[d]			0.02	0.02	0.02
Continuous[e]	69	8,032	1.31 (1.02,1.68)	1.31 (1.02,1.68)	1.30 (1.01,1.66)
**Years ½–MCL (>5 mg/L NO_3-N)**					
0	38	5,287	1.00 (Ref.)	1.00 (Ref.)	1.00 (Ref.)
< 4	5	741	0.92 (0.36,2.34)	1.01 (0.40,2.56)	0.95 (0.37,2.43)
≥ 4	26	2,004	1.81 (1.10,2.98)	1.83 (1.11,3.01)	1.80 (1.09,2.97)
p_{trend}[d]			0.02	0.02	0.02
Continuous[f]	69	8,032	1.07 (1.00,1.14)	1.07 (1.00,1.14)	1.07 (1.00,1.14)

[a] Adjusted for age.

[b] Adjusted for age, smoking status, and pack-years of smoking.

[c] Adjusted for age, smoking status, pack-years of smoking, and ln-transformed TTHM level.

[d] Estimated by modeling a continuous variable derived from the median value within each exposure category.

[e] HR per one natural log increase in concentration (mg/L).

[f] HR per one year increase in number of years >½-MCL.

Table S4. Association between average drinking water nitrate-nitrogen (NO_3-N) and bladder cancer by median total trihalomethanes (TTHM) level in the Iowa Women's Health Study (N=15,577)

	< Median TTHM (< 4.59µg/L)			≥ Median TTHM (≥ 4.59µg/L)			
**NO_3-N (mg/L)**	**Cases**	**N**	**HR[a] (95% CI)**	**Cases**	**N**	**HR[a] (95% CI)**	**$p_{interaction}$[b]**
<0.47	22	3,006	1.00 (Ref.)	7	9,67	0.96 (0.41,2.24)	
0.47-1.07	21	2,014	1.50 (0.83,2.73)	11	1,839	0.79 (0.38,1.63)	
1.08-2.97	5	1,169	0.59 (0.22,1.56)	25	2,961	1.12 (0.63,1.99)	
>2.97	10	1,233	1.12 (0.53,2.37)	29	2,388	1.64 (0.94,2.86)	0.07
	58	7,422		72	8,155		

[a] Adjusted for age, smoking status, and pack-years of smoking.

[b] Derived from a likelihood ratio test comparing fit of models with and without cross-product terms for TTHM level (<, ≥ median) and nitrate quartiles.

Table S5. Association between private well use (N=4,930) and drinking water nitrate–nitrogen (NO_3-N) levels in public water supplies (N=15,577) and bladder cancer in the Iowa Women's Health Study, stratified by vitamin C intake

	> Median Vitamin C (>191 mg/day)			≤ Median Vitamin C (≤191 mg/day)			
	**Cases**	**N**	**HR[a] (95%CI)**	**Cases**	**N**	**HR[a] (95%CI)**	$p_{interaction}$[b]
**Private well[c]**	20	2,338	2.38 (1.03,5.51)	16	2,542	0.69 (0.36,1.35)	0.02
**Average NO_3-N (mg/L)**							
<0.47	8	1,952	1.00 (Ref.)	21	2,021	2.37 (1.05,5.35)	
0.47-1.07	14	1,918	1.83 (0.77,4.36)	18	1,935	2.15 (0.93,4.94)	
1.08-2.97	20	2,026	2.38 (1.05,5.41)	10	2,104	1.07 (0.42,2.72)	
>2.97	15	1,818	2.03 (0.86,4.78)	24	1,803	3.05 (1.37,6.79)	0.27
	57	7,714		73	7,863		
**Years ½–MCL (>5 mg/L NO_3-N)**							
0	34	5,398	1.00 (Ref.)	49	5,549	1.30 (0.84,2.02)	
< 4	10	1,137	1.42 (0.70,2.88)	8	1,158	1.04 (0.48,2.24)	
≥ 4	13	1,179	1.71 (0.90,3.25)	16	1,156	2.03 (1.12,3.68)	0.27
	57	7,714		73	7,863		

[a] Adjusted for age, smoking status, and pack-years of smoking.
[b] Derived from a likelihood ratio test comparing fit of models with and without a cross-product term for vitamin C (>, ≤median) and nitrate exposure.
[c] Compared to a reference group of women in Q1 of NO_3-N on public water supplies (N=3,973).

Table S6. Association between dietary nitrate and nitrite and bladder cancer in the Iowa Women's Health Study, stratified by vitamin C intake (N=33,964)

	> Median Vitamin C (>191 mg/day)			≤ Median Vitamin C (≤191 mg/day)			
	**Cases**	**N**	**HR[a] (95%CI)**	**Cases**	**N**	**HR[a] (95%CI)**	$p_{interaction}$[b]
**Dietary nitrate (mg NO_3-N/day[c])**							
All sources							
<16.2	22	2,691	1.00 (Ref.)	45	5,776	0.90 (0.54,1.50)	
16.2-23.9	27	3,565	0.92 (0.52,1.62)	41	4,924	0.95 (0.57,1.61)	
24.0-34.2	37	4,524	0.97 (0.57,1.67)	27	3,982	0.76 (0.43, 1.35)	
>34.2	37	6,192	0.69 (0.39,1.20)	22	2,310	1.05 (0.57,1.92)	0.28
	123	16,972		135	16,992		
**Dietary nitrite (mg/day)**							
All sources							
<0.86	16	3,053	1.00 (Ref.)	47	5,397	1.55 (0.88,2.74)	
0.86-1.12	29	3,855	1.62 (0.56,3.05)	37	4,659	1.56 (0.85,2.85)	
1.13-1.43	35	4,426	1.84 (0.95,3.54)	38	4,061	1.98 (1.05,3.75)	
>1.43	43	5,638	1.94 (0.94,4.00)	13	2,875	1.00 (0.44,2.31)	0.48
	123	16,972		135	16,992		

[a] Adjusted for age, smoking status, pack-years of smoking, and ln-transformed total energy intake. Nitrate models were also adjusted for total ln-transformed dietary nitrite from all sources, and nitrite models were adjusted for total ln-transformed dietary nitrate from all sources.
[b] Derived from a likelihood ratio test comparing fit of models with and without a cross-product term for vitamin C (>, ≤median) and dietary nitrate or nitrite quartiles.
[c] NO_3 converted to NO_3-N.

Table S7. Association between dietary nitrate and nitrite and bladder cancer in the Iowa Women's Health Study, stratified by smoking status (N=33,964)

	Never smokers			Former smokers			Current smokers			
	**Cases**	**N**	**HR[a] (95% CI)**	**Cases**	**N**	**HR[a] (95% CI)**	**Cases**	**N**	**HR[a] (95% CI)**	$p_{interaction}$[c]
**Dietary Nitrate (mg NO_3-N/day[b])**										
All sources										
<16.2	34	5,368	1.00 (Ref.)	18	2,158	0.87 (0.46,1.67)	15	941	1.98 (0.97,4.08)	
16.2-23.9	34	5,607	0.92 (0.57,1.49)	21	2,113	1.05 (0.57,1.95)	13	769	1.94 (0.91,4.11)	
24.0-34.2	31	5,737	0.80 (0.48,1.31)	24	2,186	1.10 (0.61,2.02)	9	583	1.72 (0.75,3.95)	
>34.2	26	5,713	0.65 (0.38,1.11)	24	2,256	1.06 (0.58,1.96)	9	533	1.85 (0.80,4.29)	0.10
	125	22,425		87	8,713		46	2,826		
**Dietary Nitrite (mg/day)**										
All sources										
<0.86	30	4,980	1.00 (Ref.)	21	2,500	0.92 (0.49,1.74)	12	970	1.48 (0.69,3.22)	
0.86-1.12	29	5,508	0.90 (0.52,1.55)	26	2,290	1.31 (0.70,2.46)	11	716	1.97 (0.87,4.44)	
1.13-1.43	41	5,795	1.25 (0.72,2.16)	19	2,090	1.12 (0.55,2.27)	13	602	2.87 (1.27,6.51)	
>1.43	25	6,142	0.75 (0.38,1.49)	21	1,833	1.49 (0.70,3.21)	10	538	2.66 (1.05,6.75)	0.06
	125	22,425		87	8,713		46	2,826		

[a] Adjusted for age, pack-years of smoking, and ln-transformed total energy intake. Nitrate models were also adjusted for total ln-transformed dietary nitrite from all sources, and nitrite models were adjusted for total ln-transformed dietary nitrate from all sources.
[b] NO_3 converted to NO_3-N.
[c] Derived from a likelihood ratio test comparing fit of models with and without cross-product terms for smoking category and dietary nitrate or nitrite quartiles.
